# Identification of the endosomal sorting complex required for transport-I (ESCRT-I) as an important modulator of anti-miR uptake by cancer cells

**DOI:** 10.1093/nar/gku1367

**Published:** 2014-12-30

**Authors:** Timothy R. Wagenaar, Tatiana Tolstykh, Chaomei Shi, Lan Jiang, JingXin Zhang, Zhifang Li, Qunyan Yu, Hui Qu, Fangxian Sun, Hui Cao, Jack Pollard, Shujia Dai, Qiang Gao, Bailin Zhang, Heike Arlt, May Cindhuchao, Dietmar Hoffmann, Madelyn Light, Karin Jensen, Joern Hopke, Richard Newcombe, Carlos Garcia-Echeverria, Christopher Winter, Sonya Zabludoff, Dmitri Wiederschain

**Affiliations:** 1 Sanofi Oncology, Cambridge, MA 02139, USA; 2 Regulus Therapeutics, San Diego, CA 92121, USA

## Abstract

Mechanisms of unassisted delivery of RNA therapeutics, including inhibitors of microRNAs, remain poorly understood. We observed that the hepatocellular carcinoma cell line SKHEP1 retains productive free uptake of a miR-21 inhibitor (anti-miR-21). Uptake of anti-miR-21, but not a mismatch (MM) control, induces expression of known miR-21 targets (DDAH1, ANKRD46) and leads to dose-dependent inhibition of cell growth. To elucidate mechanisms of SKHEP1 sensitivity to anti-miR-21, we conducted an unbiased shRNA screen that revealed tumor susceptibility gene 101 (TSG101), a component of the endosomal sorting complex required for transport (ESCRT-I), as an important determinant of anti-proliferative effects of anti-miR-21. RNA interference-mediated knockdown of TSG101 and another ESCRT-I protein, VPS28, improved uptake of anti-miR-21 in parental SKHEP1 cells and restored productive uptake to SKHEP1 clones with acquired resistance to anti-miR-21. Depletion of ESCRT-I in several additional cancer cell lines with inherently poor uptake resulted in improved activity of anti-miR-21. Finally, knockdown of TSG101 increased uptake of anti-miR-21 by cancer cells *in vivo* following systemic delivery. Collectively, these data support an important role for the ESCRT-I complex in the regulation of productive free uptake of anti-miRs and reveal potential avenues for improving oligonucleotide free uptake by cancer cells.

## INTRODUCTION

MicroRNAs (miRNAs or miRs) are a class of evolutionarily conserved, short non-coding RNAs that play a critical regulatory role in many processes, including control of cellular development, metabolism, cell cycle and apoptosis. miRNAs exert their function through post-transcriptional regulation of mRNA stability or inhibition of translation through an interaction with the 3′UTR (1). Accumulating evidence suggests that loss of function or overexpression of miRNAs contributes to the development and progression of many common human diseases, including metabolic syndromes, heart disease and cancer (2).

A search of publicly available human genome and small RNA sequencing data yields >1500 annotated miRNA, with around 300–500 high confidence human miRNA depending on the criteria used to define a functional miRNA (3). Among one of the most intensively studied miRNAs is miR-21. Numerous studies have demonstrated miR-21 to be upregulated in a wide range of human cancers, and elevated miR-21 levels are consistently associated with poor patient prognosis (4,5). Additionally, elevated levels of miR-21 is thought to contribute to cardiovascular disease and fibrosis of the lung and kidney (6–8). Given its prominent role in human disease, therapeutic utility of miR-21 inhibition is being investigated in pre-clinical models of cancer and other diseases associated with miR-21 overexpression (9,10). A common approach to inhibit miR-21 and other miRNAs is through the use of short single-stranded oligonucleotides (anti-miRs) (11).

Anti-miRs are chemically modified oligonucleotides that functionally inhibit miRNAs through sequence-specific binding. Through this interaction, the anti-miR sequesters miRNA, thus preventing it from inhibiting its target messenger RNA and thereby restoring normal gene expression. Incorporation of various oligonucleotide modifications into the anti-miRs increases their resistance to nucleases and their affinity for target miRNAs, leading to improved *in vitro* and *in vivo* efficacy (12–14). Systemically administered anti-miRs broadly distribute within the body, with notable accumulation in the liver and kidney, and have been shown to affect miRNA function *in vivo* (15).

While the downstream effects of anti-miRs on miRNAs and their targets have been studied extensively, the precise mechanisms of anti-miR uptake into the cell remain poorly defined. Single-stranded oligonucleotides enter cells using a variety of endocytic pathways that may differ between cell types (16–18). Following endocytosis, oligonucleotides are transported through multiple vesicular compartments including early/sorting endosomes, late endosomes/multi-vesicular bodies, lysosomes and the *trans*-Golgi network prior to being released into the cytoplasm. The uptake of anti-miRs and other single-stranded oligonucleotides, including gapmer-based antisense oligonucleotides (ASO), can be broadly categorized as ‘productive’ and ‘non-productive’. Productive uptake leads to the cytoplasmic release of the oligonucleotide and interaction with its target nucleic acid, while during non-productive uptake the oligonucleotide is unable to gain access to its target, likely due to entrapment within intracellular vesicles. Productive uptake of anti-miR and ASO is observed both *in vitro* and *in vivo* and is influenced in part by the route of intracellular trafficking (19–21). However, a significant fraction of the oligonucleotides taken up by the cell enters a non-productive pathway and remain trapped within various membrane-bound vesicles. To shift the balance toward more productive uptake, a number of endosomolytic agents and targeting ligands have been explored to facilitate endosomal disruption and transport of the oligonucleotide into the cytoplasm, where it is able to interact with its intended target (22–25). Several studies have investigated the cellular factors that influence productive uptake independent of transfection or endosomolytic agents (20,26,27); however, additional data are clearly needed to have a better understanding of oligonucleotide trafficking and release.

The goal of this study was to gain additional insight into the mechanisms that govern cancer cell sensitivity to anti-miR-21. After screening a number of cancer cell lines for their ability to take up anti-miR-21, we observed that the hepatocellular carcinoma cell line SKHEP1 retained productive free uptake of anti-miR-21 and was sensitive to the anti-proliferative effects of this anti-miR *in vitro*. An unbiased shRNA screen in SKHEP1 identified tumor susceptibility gene 101 (TSG101), a component of the endosomal sorting complex required for transport (ESCRT-I), as an important modulator of anti-miR-21 free uptake. Knockdown of TSG101, along with a second ESCRT-I protein, VPS28, enhanced uptake of anti-miR-21 across multiple cancer cell lines. These effects were confirmed in an *in vivo* mouse model of anti-miR-21 uptake and demonstrate that the ESCRT-I complex has an important role in regulating productive free uptake of oligonucleotides across a range of cancer cell types.

## MATERIALS AND METHODS

### Cell culture

SKHEP1, A549 and HUCCT1 were acquired from DSMZ, ECACC and the JCRB cell bank, respectively. Short tandem repeat (STR) profiling was performed to verify cell identity. All cells were cultured in Dulbecco's modified Eagle medium (Life Technologies), supplemented with 10% Fetal bovine serum (FBS) (Invitrogen) and cultured at 37°C in a humidified incubator with 5% CO_2_.

### Anti-miR compounds

The commercial anti-miR-21 compound was purchased from Exiqon (Product number 4102261-102). All other anti-miR compounds were synthesized by Regulus Therapeutics (San Diego, CA, USA). Chemistry A is an anti-miR-21 complementary to the active site of miR-21 that contains a phosphorothioate backbone and a mixture of DNA, constrained ethyl (cEt) and 2′-*O*-methoxyethyl (2′MOE) modified nucleotides. Chemistry B is an anti-miR-21 complementary to the active site of miR-21 containing a phosphorothioate backbone and a mixture of 2′-ribo-F and 2′-MOE modified nucleotides. MM controls #1 and #2 are anti-miRs with no complementarity to the miR-21 seed sequence that contains a phosphorothioate backbone and a mixture of DNA, cEt and 2′-MOE modified nucleotides.

### shRNA screen

SKHEP1 cells were transduced at a multiplicity of infection of 0.3 with the human shRNA library (DECIPHER 27K module 1, Cellecta) in the presence of 10 μg/ml polybrene. After a 24-h infection, the culture media was supplemented with puromycin at a final concentration of 1 μg/ml. The cells were selected for 3 days, divided into two groups and treated with either 500 nM of anti-miR-21 or 500 nM MM control in the absence of transfection reagents. Cells were cultured for 13 days in the presence of the anti-miR compound, sub-culturing once on day 6. At the end of the 13 days the cells were collected and pelleted, and genomic DNA was isolated and prepared for next-generation sequencing using an Illumina HiSeq2000. The raw sequencing reads from day 13 were used to calculate the log2 ratio (anti-miR-21/MM control) for each shRNA. For each gene, shRNA were rank ordered from high to low based on the log2 ratio (anti-miR-21/MM control). Candidate genes were defined as having two or more shRNA with a log2 ratio of ≤−1.5.

### miR-21 luciferase reporter

A lentiviral miR-21 reporter based on pL/SV40/GL3 was prepared by inserting a perfectly complementary miR-21 binding site into the 3′UTR of the firefly luciferase mRNA (28).

### siRNA transfections and shRNA knockdown

siRNA and shRNA used in this study are listed in Supplementary Table S1. Lyophilized siRNA were reconstituted in nuclease-free water and used at a final concentration of 10 nM for all experiments. Lipid-mediated transfection was performed in a 6-well tissue culture plate using RNAiMAX (Life Technologies). Briefly, 9 μl of RNAiMAX was mixed with 291 μl OptiMEM^®^ (Life Technologies). This mixture was combined with 300 μl of a 100 nM siRNA mixture prepared in OptiMEM^®^. The RNAiMAX/siRNA mixture was incubated for 10 min, and the entire 600 μl was added to a single well of a 6-well tissue culture plate. Cells (6 × 10^5^ in 2400 μl of growth media) were added to the transfection mixture. At 24 h post-transfection, the cells were trypsinized and suspended at a dilution of 5 × 10^4^ cells/ml. Note that 100 μl of cell suspension was dispensed into duplicate 96-well plates. The following day, a 6-point dose–response dilution series of anti-miR was prepared and then added to the cells. The cells were treated with anti-miR for 48 h and the miR-21 luciferase reporter and cell number were quantified with Bright-Glo^TM^ (Promega) and CellTiter-Glo^®^ (Promega), respectively. Luminescence was measured using an Envision plate reader (PerkinElmer). The miR-21 luciferase reporter values were normalized to relative cell number.

### Transfection and uptake experiments for anti-miR and miR-mimic

Cells were reverse-transfected with anti-miR for a 6-point dose-response curve starting at 25 nM with 5-fold dilution steps. For the transfection, anti-miR was diluted in OptiMEM^®^ to 10× final concentration and mixed with an equal volume of RNAiMAX diluted in OptiMEM^®^. Transfections were done according to the manufacturer's recommendation, using 0.3 μl of RNAiMAX per well of 96-well plate. Twenty microliters of anti-miR/RNAiMAX mixture was added per well of 96-well plate and 80 μl of cells (5 × 10^3^–1 × 10^4^ cells/well) was added. After 24 h of incubation, 80 μl of Bright-Glo^TM^ (Promega) was added per well and incubated for 10 min. Luminescence was read using an Envision plate reader. For unassisted uptake experiments, 2.5 × 10^3^ cells were plated in 100 μl per well of a 96-well plate and allowed to attach overnight. The anti-miR compounds were prepared in growth media and 10 μl of a 10× solution was added to the cells. Typically, a 6-point dose–response with 4-fold dilution steps was prepared. The highest dose for unassisted uptake was typically 5–10 μM. Cells were incubated for 48–72 h, the luciferase signal was developed using Bright-Glo^TM^ and luminescence was read using an Envision plate reader. For monitoring endogenous miR-21 target derepression, 1 × 10^4^ cells/well of 96-well plate were transfected using RNAiMAX with either 25 nM of miR-21 mimic (Life Technologies), 25 nM of MM control mimic (Life Technologies), 25 nM of anti-miR-21 (Chemistry A) or 25 nM of MM control. Cells were lysed at 48 h post-transfection and total RNA was isolated.

### RNA isolation and real-time quantitative polymerase chain reaction (RT-qPCR)

RNA was isolated using the 96 RNeasy kit (Qiagen), following the vacuum/spin protocol without the optional DNase treatment. RNA was eluted into nuclease-free water and quantified by ultraviolet absorbance using a NanoDrop 8000 (Thermo Scientific). cDNA synthesis was performed using the High Capacity RNA to cDNA Kit (Applied Biosystems) according to the manufacturer's recommendations. The cDNA was diluted 1:4 with nuclease-free water and 1 μl was added to a 10 μl Taqman reaction mixture prepared using the TaqMan Gene Expression Master Mix (Applied Biosystems). Taqman assays were performed on a ViiA^TM^7 (Applied Biosystems) or a QuantStudio^TM^ 12K Flex Real Time PCR System (Applied Biosystems) using the following conditions: 50°C for 2 min; 95°C for 10 min; 40 cycles of 95°C for 15 s and 60°C for 1 min. Gene expression was normalized to the endogenous control GAPDH and RNU48 for mRNA and miRNA expression, respectively. Taqman primers and probes are listed in Supplementary Table S2.

### Immunoblotting

Total cell lysates were prepared in RIPA Buffer (Pierce) supplemented with HALT Protease and Phosphatase Inhibitor (Pierce). Lysates were pelleted at 12 000 × *g* for 20 min and the supernatant was collected. Protein lysates were quantified using a Bicinchoninic Acid Assay (Pierce). Protein lysates were mixed with NuPAGE LDS Sample Buffer (Life Technologies), loaded onto a 4–12% Bis-Tris gradient gel (Life Technologies), and subjected to electrophoresis in MES buffer (Life Technologies). The proteins were transferred to a nitrocellulose membrane using an iBlot gel transfer device (Life Technologies). The nitrocellulose membrane was blocked with a 5% non-fat milk solution prepared in TBS-Tween (Cell Signaling). Primary antibodies to TSG101 (1:2000, clone EPR7130(B), Millipore) and β- Actin (1:10000, AC-15, Santa Cruz) were incubated overnight at 4°C with rocking in 2% non-fat milk/TBS-Tween solution. Membranes were extensively washed and incubated with secondary antibody (1:5000, Pierce). Membranes were developed with Super Signal West Pico Chemiluminescent Substrate (Pierce) and exposed to film.

### Animal handling

All *in vivo* experiments were performed according to the institutional guidelines and approved by the Institutional Animal Care and Use Committee. shRNA expression was induced in the +DOX group 24 h prior to implantation by addition of 1 μg/ml doxycycline to the culture media, while cells in the −DOX group were left untreated. Female Severe combined immunodeficiency (SCID) mice (Charles River laboratory) in the +DOX group were switched to 400 ppm doxycycline diet (Harlan) 3 days prior to implant, while animals in the −DOX group were maintained on standard mouse diet. On the day of implant, the SKHEP1 reporter cell lines were suspended in PBS at a concentration of 1 × 10^7^ cells per milliliter, and 500 μl of the suspension was injected into the peritoneal cavity of each mouse. Luciferase imaging was performed on a Xenogen IVIS imaging system (Perkin Elmer) following intraperitoneal injection of 150 mg/kg of *D*-luciferin (Perkin Elmer) prepared in PBS. Anti-miR compounds were formulated in PBS and administered intraperitoneal injection at 25 mg/kg.

## RESULTS

### SKHEP1 cells display productive uptake of anti-miR-21

To investigate the ability of cancer cell lines to productively internalize anti-miR-21, we utilized a miR-21 luciferase reporter. This reporter contains the firefly luciferase gene under the control of an SV40 constitutive promoter and a perfectly complementary miR-21 recognition site in the 3′ UTR. In the presence of endogenous miR-21, luciferase levels are repressed, but upon introduction of anti-miR-21, luciferase levels increase, serving as a sensitive and rapid readout of miR-21 activity.

**Figure 1.**
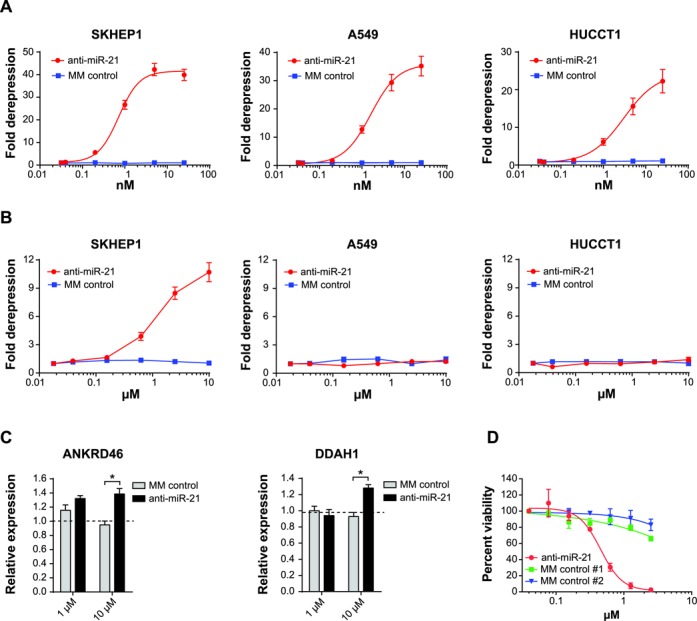
SKHEP1 exhibits productive uptake of anti-miR-21. (**A**) SKHEP1, A549 and HUCCT1 cells expressing miR-21 luciferase reporter were transfected with a dilution series of anti-miR-21 or MM control, and miR-21 luciferase reporter activity was measured at 24 h post-transfection. Mean ± SEM, *n* = 3. (**B**) Anti-miR-21 or MM control was added to the culture media of the SKHEP1, A549 and HUCCT1 miR-21 reporter cell lines. Luciferase activity was measured after 72 h of uptake. Mean ± SEM, *n* = 3. (**C**) SKHEP1 cells were treated with PBS or oligonucleotides (anti-miR-21 or MM control) at concentrations of either 1 or 10 μM. After 72 h of anti-miR treatment, total RNA was isolated and qPCR was performed to measure expression of the endogenous miR-21 targets ANKRD46 and DDAH1. Expression levels in PBS-treated samples are indicated by the dotted line. *P ≤ 0.05, Mean ± 1 SD, *n* = 2. (**D**) A dilution series of anti-miR-21 or two different MM controls was added to the culture media of SKHEP1 and relative cell number was quantified after 5 days treatment. Mean ± SEM, *n* = 3.

SKHEP1, A549 and HUCCT1 cancer cell lines were generated to stably express the miR-21 luciferase reporter. To validate these miR-21 luciferase reporter cell lines, the cells were transfected with anti-miR-21 or mismatch (MM) control oligonucleotides and luciferase activity was measured after 24 h. All three cell lines transfected with anti-miR-21 exhibited strong, dose-dependent increases in luciferase activity (Figure 1A). No increase in luciferase activity was observed upon treatment with MM control, indicating this assay was specific for miR-21 inhibition as opposed to non-specific effects associated with transfection of short oligonucleotides.

We next evaluated the uptake of anti-miR oligonucleotides in the absence of cationic lipid-based transfection by adding anti-miR-21 or MM control oligonucleotide directly to the culture media of A549, HUCCT1 and SKHEP1 miR-21 reporter cell lines and measuring miR-21 inhibition after 72 h. Treatment of SKHEP1 cells with anti-miR-21, but not with MM control, showed a dose-dependent increase in luciferase activity (Figure 1B). In contrast, both A549 and HUCCT1 displayed limited productive free uptake of anti-miR compound. Compared to transfection-based delivery, unassisted uptake had slower kinetics and required higher doses of anti-miR-21, which is consistent with earlier reports using single-stranded anti-sense oligonucleotide (20).

In addition to the miR-21 luciferase reporter, we examined the effects of unassisted anti-miR uptake on several endogenous targets of miR-21 in SKHEP1. Both ANKRD46 and DDAH1 are putative miR-21 target genes that possess an octamer sequence motif complementary to miR-21 within their 3′UTRs (29,30). Transfection of SKHEP1 cells with anti-miR-21 resulted in significant derepression of ANKRD46 and DDAH1 levels (Supplementary Figure S1A). As an additional validation, SKHEP1 cells were transfected with a chemically modified double-stranded RNA designed to mimic mature miR-21 activity. Upregulation of miRNA-21 activity would be expected to decrease expression of putative miRNA target genes. Accordingly, expression of both ANKRD46 and DDAH1 was reduced by miR-21 mimic expression, thus confirming both genes are directly regulated by miR-21 (Supplementary Figure S1B). To measure the effects of unassisted uptake on endogenous miR-21 target genes, SKHEP1 cells were treated with anti-miR-21 or MM control for 72 h in the absence of transfection reagents and expression of ANKRD46 and DDAH1 was measured by qPCR. Treatment with anti-miR-21, but not with a MM control, increased expression of ANKRD46 and DDAH1 in SKHEP1 cells (Figure 1C). Furthermore, anti-miR-21 treatment produced a strong, dose-dependent reduction in cell viability (Figure 1D), consistent with suppression of the well-known growth-promoting function of miR-21 (31). Collectively, these results demonstrate that SKHEP1 cells are capable of productive free uptake of anti-miR-21, leading to endogenous target derepression and loss of cell viability over time. Therefore, SKHEP1 is a useful cellular model to study the mechanisms governing sensitivity of cancer cells to anti-miR-21 compounds.

### The ESCRT-I complex modulates uptake of anti-miR-21

**Figure 2.**
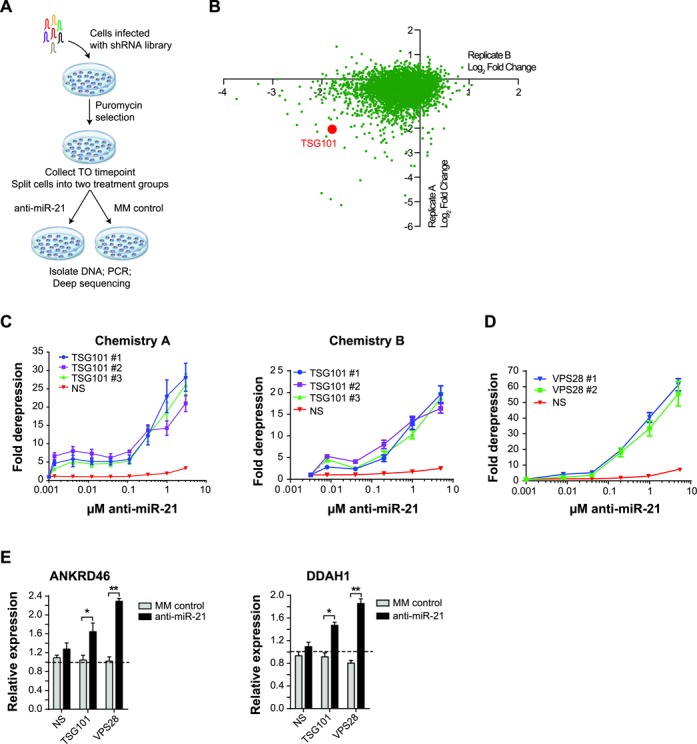
The ESCRT-I complex regulates uptake of anti-miR-21. (**A**) SKHEP1 cells were infected with an shRNA library and selected for stable transduction. At 3 days post-transduction, an aliquot of cells was collected and saved as a T0 time point. The remaining cells were split into two treatment groups and cultured for 13 days with either anti-miR-21 or MM control. At the end of 13 days, the cells were collected; genomic DNA was extracted and used as a template for PCR amplification of shRNA barcode which was sequenced on an Illuminia HiSeq2000. Two independent biological replicates of shRNA screen were performed. (**B**) Scatter plot of **l**og2 fold change (anti-miR-21 sequencing reads/MM control sequencing reads) from biological Replicate A and biological Replicate B. On average each gene was targeted by 5–6 shRNA. For each gene, the shRNA were ordered from high to low based on the log2 ratio (anti-miR-21/MM control). The value of the second lowest shRNA ratio for each gene is plotted with Replicate A on the ordinate and Replicate B on the abscissa. (**C**) SKHEP1 miR-21 luciferase cells were transfected with siRNA targeting non-silencing (NS), TSG101-siRNA#1, TSG101-siRNA#2 and TSG101-siRNA#3 or (**D**) siRNA targeting NS, VPS28-siRNA#1 and VPS28-siRNA#2. At 48 h post-transfection anti-miR-21 Chemistry A or Chemistry B was added to the culture media, and the cells were incubated for an additional 48 h, followed by measurement of miR-21 luciferase reporter activity. Luciferase expression was normalized to cell number and expressed as fold derepression compared to cells treated with media only. Mean ± SEM, *n* = 3. (**E**) SKHEP1 cells were transfected with siRNA targeting NS, TSG101 or VPS28. After 24 h knockdown with siRNA, the cells were treated by adding PBS, 1 μM anti-miR-21 or 1 μM MM control to the culture media. Following 72 h anti-miR treatment, RNA was isolated and expression of ANKRD46, DDAH1 was performed. Expression levels of PBS-treated samples are indicated by the dotted line. Data are plotted as Mean ± 1 SD, *n* = 2. *P ≤ 0.05, **P ≤ 0.01.

The free uptake of anti-miR-21 by SKHEP1 provided a cell model to explore and identify genes which modulate sensitivity to anti-miR-21. To that end, an unbiased pooled shRNA screen was conducted in the SKHEP1 cell line with a particular focus on identifying shRNAs that sensitized cells to the anti-proliferative effects of miR-21 inhibition (Figure 2A). To perform the screen, SKHEP1 cells were transduced with an shRNA library targeting ∼5000 well-annotated genes with established roles in cell signaling and human disease. After stable transduction, the cells were split into two groups and anti-miR-21 or MM control was added to the culture media for the duration of the 13 days. Next-generation sequencing was performed to identify genes targeted by two or more shRNA. In particular, we focused on genes which were strongly depleted (−1.5 log^2^) in both biological replicates by anti-miR-21 treatment, but not MM control anti-miR. Using this criteria 11 candidates emerged with tumor susceptibility gene 101 (TSG101) among one of the top genes (Figure 2B and Supplemental Data S1).

TSG101 is part of the ESCRT-I (32). ESCRT-I functions with the ESCRT-0, -II, -III in the sorting and trafficking of ubiquitylated cargo (33). Together the ESCRT complexes are critical mediators in the biogenesis of multivesicular bodies (MVB). The established role of TSG101 in endosomal trafficking, combined with the knowledge that single-stranded oligonucleotides are taken up into the cell through an endocytic process, prompted us to examine in more detail the role of TSG101 in the uptake of anti-miR-21.

Transfection of SKHEP1 miR-21 reporter with three independent siRNAs targeting TSG101 led to robust depletion of TSG101 mRNA and protein (Supplementary Figure S2A and B). The knockdown of TSG101 significantly improved productive free uptake of anti-miR-21 as indicated by the more than 20-fold increase in the activity of miR-21 luciferase reporter (Figure 2C). The effects of TSG101 knockdown were not limited to a specific anti-miR, as the uptake of two additional chemically distinct anti-miR-21 was similarly improved by TSG101 siRNA treatment (Figure 2C and Supplementary Figure S2C). Importantly, knockdown of TSG101 had limited effects on the level of mature miR-21 (Supplementary Figure S2D).

The ESCRT-I complex is a hetero-tetramer consisting of TSG101, VPS28, VPS37 (A–D) and MVB12 (A, B) (34). Identification of TSG101 in our screen suggested a broader role of the entire ESCRT-I complex in the regulation of uptake of anti-miR-21 by cancer cells. However, within the original screening library no shRNAs were present which targeted any of the other ESCRT-I components. Therefore, to test a broader role of the entire ESCRT-I complex in the regulation of uptake of anti-miR we knocked down a second ESCRT-I subunit, VPS28, in SKHEP1 cells. Strikingly, reduction in VPS28 levels using two independent siRNAs also increased anti-miR-21 uptake as measured by the miR-21 luciferase reporter (Figure 2D). On-target knockdown of VPS28 was confirmed by qRT-PCR (Supplementary Figure S2E). In addition to significantly increasing anti-miR-21-dependent miR-21 reporter activity, knockdown of TSG101 or VPS28 similarly improved anti-miR-21-mediated derepression of two endogenous miR-21 targets ANKRD46 and DDAH1 (Figure 2E). Collectively, these results suggest that the ESCRT-I complex has an important regulatory role in the productive free uptake of single-stranded anti-miRs.

### ESCRT-I knockdown restores uptake to anti-miR-21 resistant SKHEP1 clones

**Figure 3.**
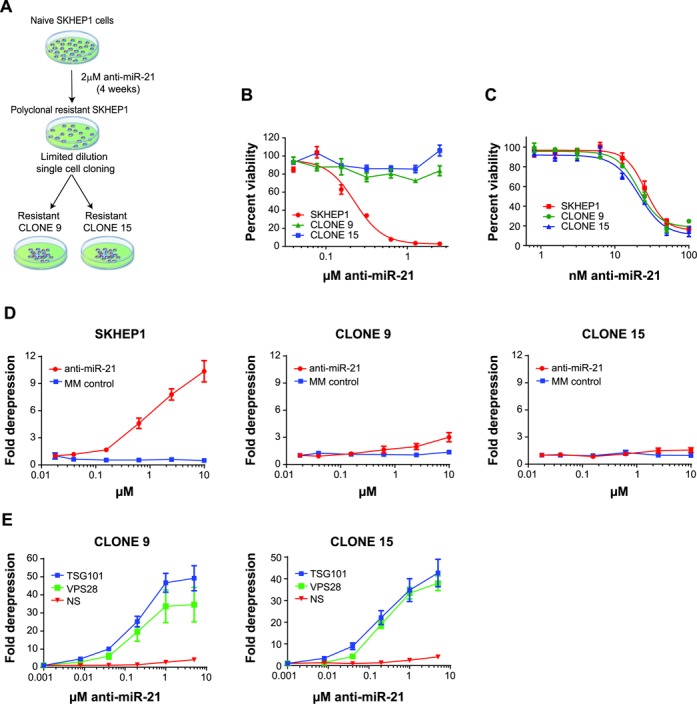
Knockdown of the ESCRT-I complex restores productive uptake in anti-miR-21 resistant clones. (**A**) SKHEP1 sensitive to anti-miR-21 were treated for 4 weeks with 2 μM anti-miR-21, subculturing the cells as needed. After 4 weeks, a polyclonal resistant population capable of growing in 2 μM anti-miR-21 was obtained. Limiting dilution cloning was performed to obtain resistant CLONE9 and CLONE15. (**B**) The SKHEP1, CLONE9 and CLONE15 cell lines were treated with a dilution series of anti-miR-21. The anti-miR-21 compound was added to the culture media and cells were grown for 5 days prior to quantification of cell number. (**C**) SKHEP1, CLONE9 and CLONE15 were transfected with a dilution series of anti-miR-21 and cell viability was quantified at 72 h post-transfection. (**D**) Anti-miR-21 or MM control was added to the culture media in the absence of cationic transfection lipid to SKHEP1, CLONE9 and CLONE15 expressing the miR-21 luciferase reporter. The cells were treated for 72 h with anti-miR and then luciferase activity was measured. (**E**) CLONE9 and CLONE15 were transfected with siRNA targeting non-silencing (NS), TSG101 or VPS28. After 48 h siRNA knockdown, anti-miR-21 was added to culture media and luciferase activity was measured after 48 h treatment. For all experiments data are plotted as Mean ± SEM, *n* = 3.

While knockdown of the ESCRT-I complex was able to enhance uptake of anti-miR in SKHEP1, a cell line already capable of productive free uptake of anti-miR, it was not known if depletion of TSG101 or VPS28 could restore productive uptake to a cell line with poor productive oligonucleotide uptake. To address this question we examined the role of the ESCRT-I complex in several SKHEP1 cell clones (CLONE9 and CLONE15) that had lost productive uptake of anti-miR. These cell clones were generated by long-term culture in the presence of high dose of anti-miR-21, followed by limited dilution cloning (Figure 3A). Resistance to anti-miR occurred at the level of productive free uptake of anti-miR-21 as CLONE9 and CLONE15 were refractory to free uptake-mediated anti-proliferative effects of anti-miR-21 (Figure 3B). In contrast, delivery of anti-miR-21 by lipid-mediated transfection to the parental SKHEP1, as well as resistant CLONE9 and CLONE15, showed a similar dose-dependent reduction in cell viability across the three cell lines (Figure 3C). To further validate that resistance to anti-miR-21 occurred at the level of uptake, we introduced miR-21 reporter into resistant SKHEP1 clones. Upon addition of anti-miR-21 to the culture media, minimal increase in luciferase activity was observed in both CLONE9 and CLONE15 (Figure 3D). This was in contrast to strong derepression of miR-21 reporter in CLONE9 and CLONE15 cell lines upon transfection-mediated delivery of anti-miR-21 (Supplementary Figure S3A).

No increase in mature miR-21 transcript was noted in CLONE9 and CLONE15, suggesting that overexpression of the target was not responsible for the resistance to anti-miR-21 compound (Supplementary Figure S3B). In addition, no changes in the expression of TSG101 were noted between the parental SKHEP1, CLONE9 and CLONE15 (Supplementary Figure S3C and D). Collectively, these data suggest CLONE9 and CLONE15 have lost the productive uptake of anti-miR.

Since our data showed that ESCRT-I is an important regulator of uptake in the parental SKHEP1 cell, we aimed to investigate its role in the resistant cell lines. To study this, we depleted TSG101 or VPS28 in CLONE9 and CLONE15 using siRNAs and confirmed strong target knockdown of both genes (Supplementary Figure S3E and F). Remarkably, knockdown of either TSG101 or VPS28 restored productive free uptake in both resistant CLONE9 and CLONE15 (Figure 3E). These results indicate that the loss of productive uptake in SKHEP1 can be overcome by the knockdown of the ESCRT-I components, and adds further evidence for the role of ESCRT-I in the regulation of productive transport of anti-miR-21 into cancer cells.

### The ESCRT-I complex regulates oligonucleotide uptake across multiple cancer cell lines

**Figure 4.**
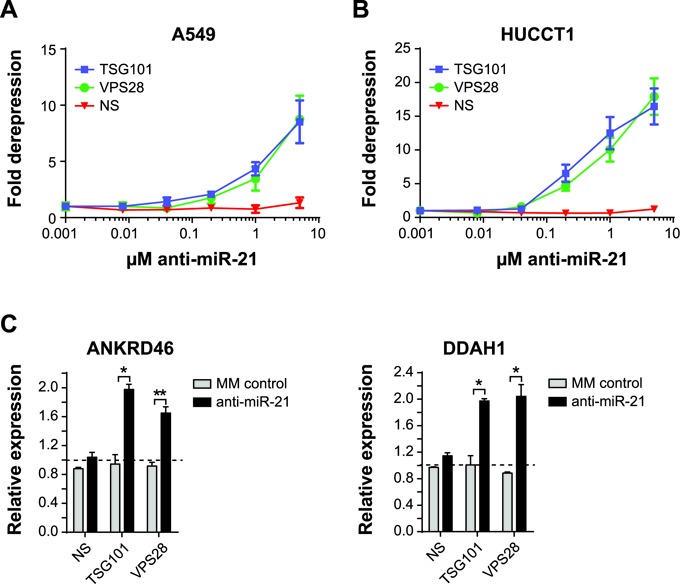
ESCRT-I restores anti-miR-21 uptake in cancer cell lines with inherently poor uptake. (**A**) A549 or (**B**) HUCCT1 cells expressing the miR-21 luciferase were transfected with siRNA targeting non-silencing (NS), TSG101 or VPS28. After 48 h siRNA knockdown, anti-miR-21 was added to culture media for an additional 48 h and then luciferase activity was measured. Mean ± SEM, *n* = 3. (**C**) A549 cell line was transfected with siRNA targeting NS, TSG101 or VPS28. At 24 h post-transfection the cells were treated by adding PBS, 1 μM anti-miR-21 or 1 μM MM control to the culture media. Following 72 h anti-miR treatment, RNA was isolated and quantitative PCR was performed to measure expression of ANKRD46, DDAH1. The dotted line represents expression of PBS-treated samples. Mean ± SD, *n* = 2. *P ≤ 0.05, **P ≤ 0.01.

Since depletion of TSG101 and VPS28 resulted in restoration of uptake in SKHEP1 anti-miR-21 resistant clones, we asked if a similar effect could be achieved in additional cancer cells lines with inherently poor free uptake of oligonucleotides. To this end, A549 and HUCCT1 miR-21 reporter cell lines were transfected with siRNAs targeting TSG101 and VPS28. Robust knockdown of both TSG101 and VPS28 was confirmed in both cell lines (Supplementary Figure S4A and B). Following knockdown of ESCRT-I components, anti-miR-21 was added to the culture media and the luciferase activity of the miR-21 reporter was monitored to quantify miR-21 inhibition. Inhibition of the ESCRT-I complex by depletion of TSG101 or VPS28 led to strong dose-dependent increase in free uptake for both A549 and HUCCT1 cell lines (Figure 4A and B).

The effect of ESCRT-I knockdown on unassisted uptake of anti-miR-21 was also evaluated by measuring expression of the endogenous miR-21 targets ANKRD46 and DDAH1 in A549 cells. As observed in SKHEP1 cells, both ANKRD46 and DDAH1 were found to be directly modulated by miR-21 in A549, as judged by the effects of transfection with anti-miR-21 and miR-21 mimic (Supplementary Figure S5A and B). Consistent with the miR-21 luciferase reporter results in A549 cells, no significant derepression of ANKRD46 or DDAH1 was observed upon anti-miR-21 addition to A549 cells treated with non-specific siRNA, thus confirming at the level of endogenous targets that this cell line has a limited ability to take up anti-miR compounds. In sharp contrast, A549 transfected with TSG101 or VPS28 siRNA responded to anti-miR-21 treatment by significantly upregulating both ANKRD46 and DDAH1 (Figure 4C). Importantly, no changes in gene expression were observed in cells treated with MM control anti-miR. These data indicate the ESCRT-I complex is an important regulator of anti-miR uptake across multiple cancer cell lines.

### TSG101 regulates productive uptake *in vivo*

**Figure 5.**
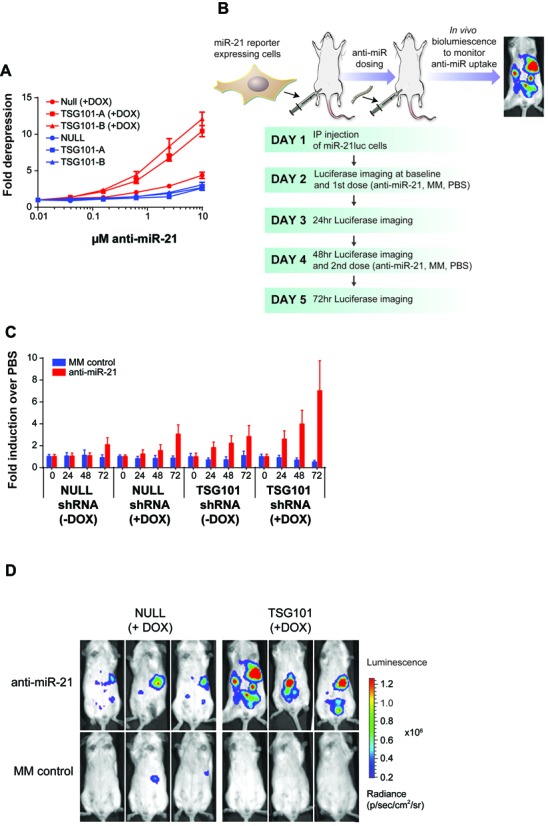
Knockdown of TSG101 improves anti-miR-21 uptake *in vivo*. (**A**) SKHEP1-miR-21 reporter cells stably expressing shRNA that target TSG101-shRNA-A, TSG101-shRNA-B and NULL were treated with doxycycline (+DOX) to induce expression of shRNA or left untreated. After 24 h knockdown, anti-miR-21 was added to the culture media and luciferase activity was measured after 72 h treatment. Mean ± SEM, *n* = 3. (**B**) Overview of *in vivo* experiment to monitor anti-miR uptake. SKHEP1miR-21 reporter cells harboring NULL or TSG101 inducible shRNA were injected into the peritoneal cavity of female immunocompromised mice. After 24 h, mice were treated with MM control or anti-miR-21 on day 2 and day 4. A baseline whole body bioluminescent imaging was performed on day 2, prior to the first dose, and then on days 3, 4 and 5, to monitor anti-miR uptake. (**C**) SKHEP1 cells co-expressing miR-21 reporter and doxycycline inducible shRNA were left untreated (−DOX) or treated with doxycycline to induce expression of shRNA (+DOX) prior to implantation into mice as described above. Animals were dosed with anti-miRs and whole body bioluminescence was used to monitor anti-miR activity *in vivo*. Luciferase values were normalized to PBS treatment group (data shown as fold luciferase induction over PBS). Mean ± SEM, *n* = 8 per group. (**D**) Representative luciferase images for 3 animals from the 72-h time point for NULL (+DOX) and TSG101 (+DOX) groups treated with anti-miR-21 or MM control.

Knockdown of TSG101 improved productive uptake of anti-miR-21 across multiple cancer cell types in tissue culture experiments, yet it was unclear if TSG101 also has an important role in regulating oligonucleotide uptake *in vivo*. To investigate anti-miR uptake *in vivo*, we generated an SKHEP1 cell line stably expressing both a miR-21 luciferase reporter and an inducible shRNA targeting TSG101. The inducible shRNA allowed knockdown of TSG101 *in vivo*, and the miR-21 reporter facilitated the measurement of productive uptake of anti-miR-21 by monitoring *in vivo* bioluminescence. Doxycycline-inducible knockdown of TSG101 was confirmed by RT-qPCR *in vitro* (Supplementary Figure S6). Consistent with the siRNA-based results, knockdown of TSG101 with two different shRNAs resulted in increased anti-miR-21 uptake as measured by the miR-21 reporter in cultured cells. Importantly, this increase was observed upon doxycycline induction only of TSG101 shRNA and not the NULL shRNA control (Figure 5A).

To monitor the effect of TSG101 on anti-miR uptake *in vivo*, SKHEP1-miR-21 luciferase cells expressing either TSG101 or NULL shRNA were injected into the peritoneal cavity of immunocompromised mice and allowed to establish for 24 h. Baseline *in vivo* bioluminescence was measured the following day, prior to the first anti-miR treatment via intraperitoneal injection. Animals were given the second dose of anti-miR-21, MM control or PBS 72 h after implantation, while the uptake of anti-miR was continuously monitored by daily luciferase imaging up to day 5 of the study (Figure 5B). Systemic treatment with anti-miR-21, but not with MM control, increased activity of miR-21 luciferase reporter over time, with the luciferase signal peaking at 72 h after the first dose of anti-miR compound. These results are consistent with the hypothesis that productive uptake of anti-miR does take place *in vivo*. Similar to the results obtained in cell culture experiments, knockdown of TSG101 significantly improved uptake of anti-miR-21 in our animal model (Figure 5C). No increase in luciferase signal was noted for animals treated with MM control. Representative images of the whole body luminescence assessment are shown in Figure 5D. Collectively, these results support the hypothesis that TSG101 regulates anti-miR-21 uptake by cancer cells both *in vitro* and *in vivo*.

## DISCUSSION

RNA therapeutics, including anti-miRs and ASO, are emerging as important treatment modalities for cancer. However, our understanding of the precise mechanisms that govern cellular uptake and trafficking of oligonucleotide-based therapeutics remains incomplete. This study aimed to elucidate molecular determinants of cancer cell sensitivity to potent and specific anti-miR-21 compounds. Our results highlight a novel role for the ESCRT-I complex in the regulation of productive uptake of anti-miR oligonucleotides by cancer cells. TSG101, one of the major ESCRT-I constituents, was identified in an unbiased, rigorously controlled shRNA depletion screen in cancer cells treated with anti-miR-21 compound. We further expanded these findings to another component of ESCRT-I complex, VPS28.

In subsequent tissue culture-based experiments, knockdown of either TSG101 or VPS28 consistently led to a significant improvement in anti-miR-21 free uptake as indicated by both miR-21 luciferase reporter and derepression of endogenous miR-21 target genes. In addition to TSG101 and VPS28, we investigated the potential role of several additional ESCRT-I components (MVB12A, VPS37A, VPS37B and VPS37D) on anti-miR uptake. Despite strong knockdown, we observed limited improvement in anti-miR-21 uptake (data not shown). In part, this may be explained by functional redundancy of the multiple isoforms of VPS37 (A, B, C, D) and MVB12 (A, B). Complete knockdown of each isoforms may be required to fully impair ESCRT-I function. In addition, combined knockdown of both TSG101 and VPS28 did not lead to further enhancement of anti-miR-21 uptake (data not shown), likely suggesting that knockdown of either TSG101 or VPS28 alone was sufficient to impair ESCRT-I function. Importantly, three distinct siRNAs and two independent shRNAs were used for TSG101, strongly suggesting that the effect of TSG101 depletion is on-target. Our data show that depletion of ESCRT-I can reverse acquired resistance to anti-miR-21, as well as improve anti-miR uptake in cancer cells with inherently poor uptake properties. Therefore, the ESCRT-I complex likely functions as an important negative regulator of productive uptake of oligonucleotides in cancer cells.

**Figure 6.**
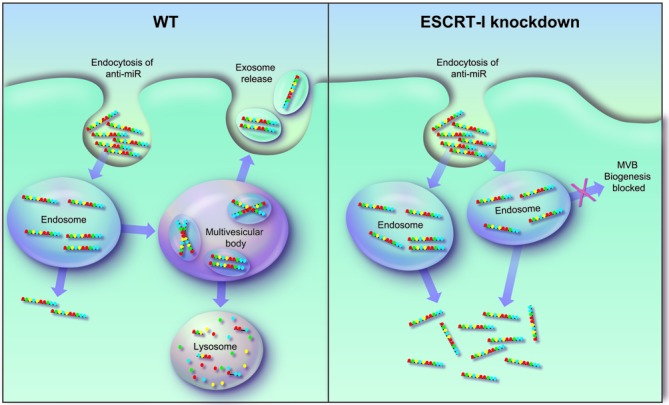
Proposed model for ESCRT-I regulation of anti-miR uptake. **LEFT:** Under normal WT conditions, anti-miRs enter cells through an endocytic process and are taken up into endosomes. Anti-miR can be transported through a productive pathway leading to release into the cytoplasm or, alternatively, can undergo non-productive uptake in which the anti-miR is shuttled to the MVB and lysosome for degradation or is recycled out of the cell. **RIGHT:** Under conditions in which the ESCRT-I complex is non-functional, MVB biogenesis is impaired, preventing transport of anti-miR through non-productive lysosome or recycling pathways. Instead, anti-miR is transported through the productive pathway, leading to improved release into the cytoplasm.

Several potential hypotheses arise as to the mechanism by which the ESCRT-I complex modulates anti-miR uptake. Endocytic vesicles loaded with anti-miR could be directed by the ESCRT-I pathway toward the MVB and ultimately transported to the lysosome for degradation. Depletion of TSG101 is known to impair biogenesis of the MVB and leads to multiple endosomal sorting defects including the disruption of transport to the lysosome (35). Therefore, in the absence of the ESCRT-I complex, anti-miR containing vesicles may be shunted away from non-productive pathways and transported through an alternative more productive pathway (Figure 6). In support of our hypothesis, a recent study using lipid nanoparticle (LNP)-encapsulated siRNA, revealed that depletion of the Niemann-Pick type C1 protein leads to endosomal retention of the LNP and enhanced cytoplasmic release of the siRNA (36). Collectively, these observations highlight the importance of vesicular trafficking to the pharmacological activity of therapeutic oligonucleotides.

While in this study we only investigated the effects of ESCRT-I complex depletion on uptake of anti-miR-21 compounds, it is plausible that ESCRT-I plays a similar role in the trafficking of other single-stranded RNA therapeutics, such as anti-sense oligonucleotides. In addition, the role of ESCRT-I in oligonucleotide trafficking in normal tissues, such as liver and kidney, warrants further investigation.

While our data suggest that modulation of ESCRT-I might improve *in vivo* efficacy of miRNA inhibitors, the ESCRT-I complex does not have known enzymatic activity, and is thus not easily druggable. Additional *in vitro* and *in vivo* studies will be needed in pre-clinical models of cancer and other diseases to identify strategies for pharmacological modulation of the ESCRT-I complex.

In summary, our study reveals a novel role of the ESCRT-I complex in the uptake of anti-miRs and highlights the opportunity to improve oligonucleotide uptake by modulating the cellular factors involved in this dynamic and complex process.

## SUPPLEMENTARY DATA

Supplementary Data are available at NAR Online.

SUPPLEMENTARY DATA
