# Supplementary material for: Identification of the endosomal sorting complex required for transport-I (ESCRT-I) as an important modulator of anti-miR uptake by cancer cells
Source: Nucleic Acids Res. 2014 Dec 30;43(2):1204–15. doi: 10.1093/nar/gku1367 (PMC4333411; doi:10.1093/nar/gku1367)
Supplement: SUPPLEMENTARY DATA [file supp_43_2_1204__index.html]

Identification of the endosomal sorting complex required for transport-I (ESCRT-I) as an important modulator of anti-miR uptake by cancer cells — Identification of the endosomal sorting complex required for transport-I (ESCRT-I) as an important modulator of anti-miR uptake by cancer cells — Identification of the endosomal sorting complex required for transport-I (ESCRT-I) as an important modulator of anti-miR uptake by cancer cells — SUPPLEMENTARY DATA 

# Identification of the endosomal sorting complex required for transport-I (ESCRT-I) as an important modulator of anti-miR uptake by cancer cells

## SUPPLEMENTARY DATA

**Files in this Data Supplement:**

- SUPPLEMENTARY DATA
- SUPPLEMENTARY DATA
