# Supplementary material for: Identification of the endosomal sorting complex required for transport-I (ESCRT-I) as an important modulator of anti-miR uptake by cancer cells
Source: Nucleic Acids Res. 2014 Dec 30;43(2):1204–15. doi: 10.1093/nar/gku1367 (PMC4333411; doi:10.1093/nar/gku1367)

SUPPLEMENTARY FIGURE LEGENDS

Supplementary Figure 1. ANKRD46 and DDAH1 are endogenous miR-21 targets in SKHEP1 cells. SKHEP1 cells were transfected with **(A)** MM control, anti-miR-21; **(B)** MM mimic or miR-21 mimic. At 48 hours post-transfection RNA was isolated and qPCR was performed to analyze mRNA levels of ANKRD46, DDAH1. Mean ±1 SD, n=2.

Supplementary Figure 2. Knockdown of ESCRT-I and its effect on miR-21 levels. A. SKHEP1 cells were transfected with siRNA to non-silencing (NS), TSG101 siRNA#1, TSG101 siRNA #2, or TSG101 siRNA#3. The RNA was isolated at 48 hours post-transfection and quantitative PCR was performed to analyze mRNA levels of TSG101. Mean ±1 SD. n=2. **B.** Protein lysates were collected from SKHEP1 cells after 72 hours siRNA knockdown with NS, TSG101 siRNA#1, TSG101 siRNA #2, or TSG101 siRNA#3. Lysates were run on SDS-PAGE and western blot analysis was performed to analyze protein levels of TSG101 or β-actin. **C.** SKHEP1 cells were transfected with siRNA targeting NS or TSG101. At 48 hrs post-transfection cells were treated with 10 μM anti-miR-21 as indicated. Luciferase activity was measured after 48 hrs of free uptake. Mean±SEM, n=3. **D.** SKHEP1 cells were transfected with siRNA targeting NS or TSG101 siRNA#1, TSG101 siRNA #2, or TSG101 siRNA#3. At 48 hours post-transfection RNA was isolated and qPCR was performed to measure miR-21 levels. Mean±1 SD, n=2. **E.** At 48 hours post-transfection, RNA was isolated from SKHEP1 cells treated with NS siRNA, VPS28 siRNA#1 or VPS28 siRNA #2. Quantitative PCR was performed to analyze mRNA levels of VPS28. Mean±1 SD, n=2.

Supplementary Figure 3. Evaluation of miR-21 reporter cell lines, mature miR-21 and TSG101 levels and confirmation of gene knockown in anti-miR-21-resistant cell clones. A. SKHEP1, CLONE9 and CLONE15 expressing the miR-21 luciferase reporter were transfected with MM control or anti-miR-21 and luciferase activity was measured at 24 hours post-transfection. Mean± SEM, n=3. **B.** Total RNA was isolated from parental SKHEP1, anti-miR-21 resistant CLONE9, and CLONE15. RNA was analyzed by qPCR to assess levels of miR-21 and **C.** mRNA levels of TSG101. Mean± 1 SD, n=2. **D.** Immunoblot analysis of the protein lysates from parental SKHEP1, anti-miR-21 resistant CLONE9, and CLONE15 was performed to assess levels of TSG101 and β-actin. **E-F.** CLONE9, and CLONE15 were transfected with non-silencing (NS) siRNA or siRNA targeting TSG101 or VPS28. RNA was isolated at 48 hours post-transfection and expression of TSG101 and VPS28 was determined by qPCR. Mean± 1 SD, n=2.

Supplementary Figure 4. ESCRT-I knockdown in A549 and HUCCT1 cells. A549 and HUCCT1 cells were transfected with non-silencing (NS) siRNA or siRNA targeting TSG101 **(A)** or VPS28 **(B)**. RNA was isolated after 48 hours and mRNA levels for TSG101 and VPS28 was determined by qPCR. Mean, ± 1 SD, n=2.

Supplementary Figure 5. ANKRD46 and DDAH1 are endogenous miR-21 targets in A549 cells. A-B. A549 cells were transfected with MM control, anti-miR-21, MM mimic or miR-21 mimic. At 48 hours post-transfection RNA was isolated and qPCR was performed to analyze mRNA levels of ANKRD46 and DDAH1. Mean, ± 1 SD, n=2.

Supplementary Figure 6. shRNA-mediated TSG101 knockdown in SKHEP1 miR-21 reporter cells. SKHEP1 miR-21 luciferase cells were stably transduced with tetracycline inducible NULL or two different shRNA targeting TSG101 (TSG101-A, TSG101-B). Cells were treated with doxycycline (+DOX) to induce expression of shRNA or left untreated (NO DOX). After 72 hours of treatment, RNA was isolated and qPCR was performed to expression of TSG101. Mean ± 1 SD, n=2.

Supplementary Table 1

siRNA reagents					
**siRNA**	**Vendor**	**Catalogue #**	**siRNA ID#**	**Sense (5'-3')**	**Anti-Sense (5'-3')**
VPS28 siRNA#1	Ambion	4427037	s27579	GAAGUGAAGUUGUACAAGATT	UCUUGUACAACUUCACUUCCT
VPS28 siRNA#2	Ambion	4427037	s27577	AAUCAGCUCUAUUGACGAATT	UUCGUCAAUAGAGCUGAUUTC
TSG101 siRNA#1	Ambion	4427037	s14439	GAAAAAGGGUCACCAGAAATT	UUUCUGGUGACCCUUUUUCAG
TSG101 siRNA#2	Ambion	4427037	s14440	CUGUCAAUGUUAUUACUCUTT	AGAGUAAUAACAUUGACAGTT
TSG101 siRNA#3	Ambion	4427037	s14441	GAGACCUAACUGUACGUGATT	UCACGUACAGUUAGGUCUCTG
NS	ThermoScientific	D-001810-10-20			
**shRNA reagents**					
**shRNA**	**Clone ID**	**Target Sequence**	**Oligonucleotide Sequence**		
TSG101-A	TRCN0000007564	GCAGAGCTCAATGCCTTGAAA	Forward	CCGGGCAGAGCTCAATGCCTTGAAACTCGAGTTTCAAGGCATTGAGCTCTGCTTTTTG	
			Reverse	AATTCAAAAAGCAGAGCTCAATGCCTTGAAACTCGAGTTTCAAGGCATTGAGCTCTGC	
TSG101-B	TRCN0000379499	CAGTCTTCTCTCGTCCTATTT	Forward	CCGGCAGTCTTCTCTCGTCCTATTTCTCGAGAAATAGGACGAGAGAAGACTGTTTTTG	
			Reverse	AATTCAAAAACAGTCTTCTCTCGTCCTATTTCTCGAGAAATAGGACGAGAGAAGACTG	

Supplementary Table 2

Taqman Assay			
**Gene**	**Vendor**	**Catalogue #**	**Assay ID**
huGAPDH	Life Technologies	4326317E	
VPS28	Life Technologies	4331182	hs00211938_m1
TSG101	Life Technologies	4331182	hs01121709_m1
ANKRD46	Life Technologies	4331182	Hs01569215_m1
DDAH1	Life Technologies	4331182	Hs00201707_m1
hsa-miR-21-5p	Life Technologies	4440887	000397
RNU48	Life Technologies	4440887	0010006

Supplementary Figure 1

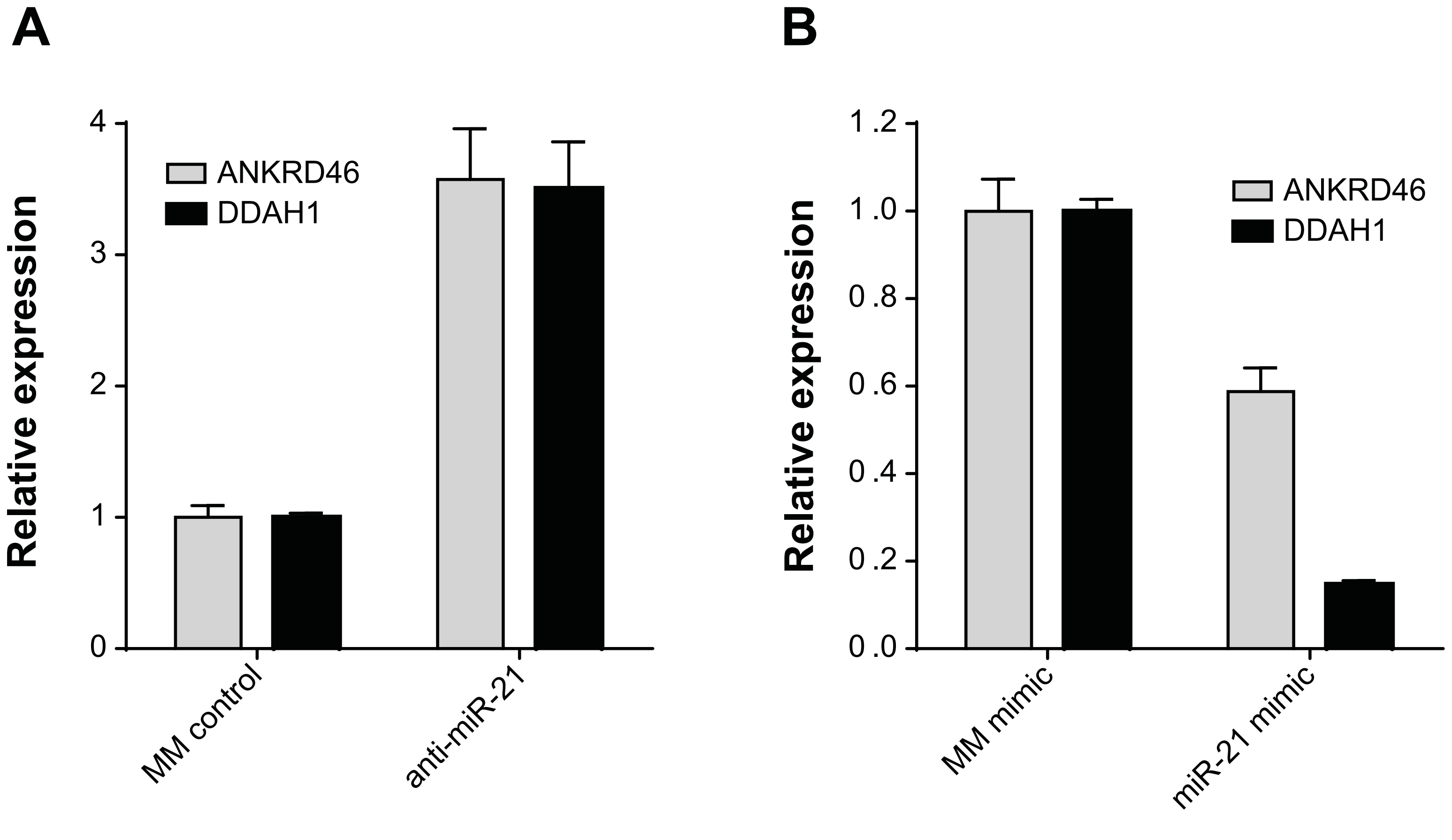

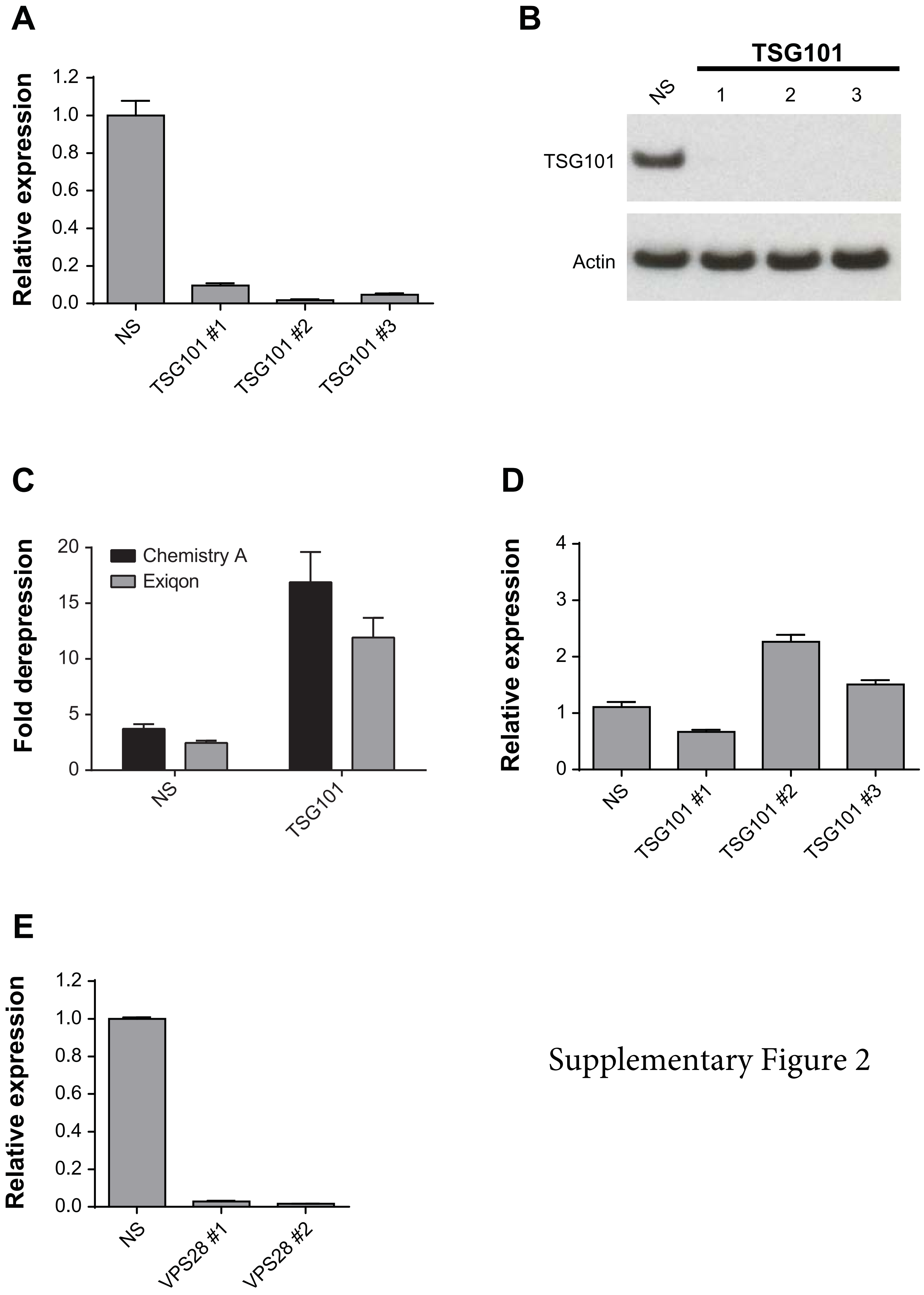

Supplementary Figure 2

Supplementary Figure 3

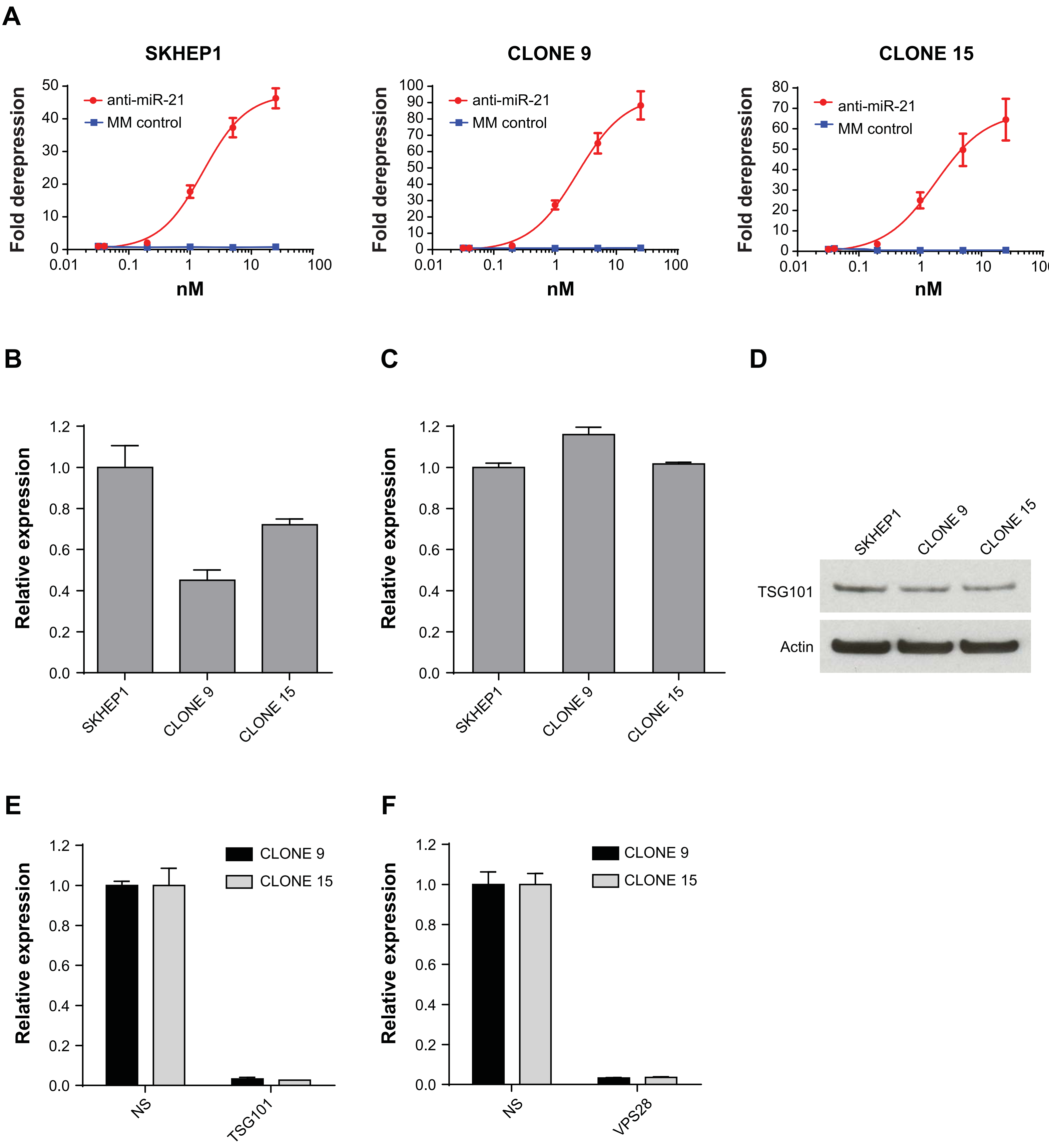

Supplementary Figure 4

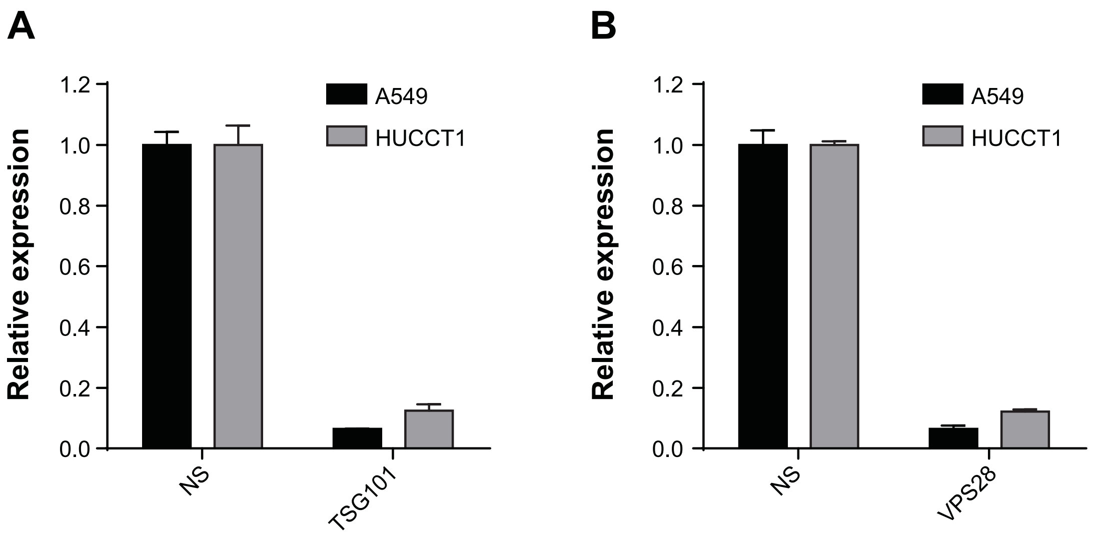

Supplementary Figure 5

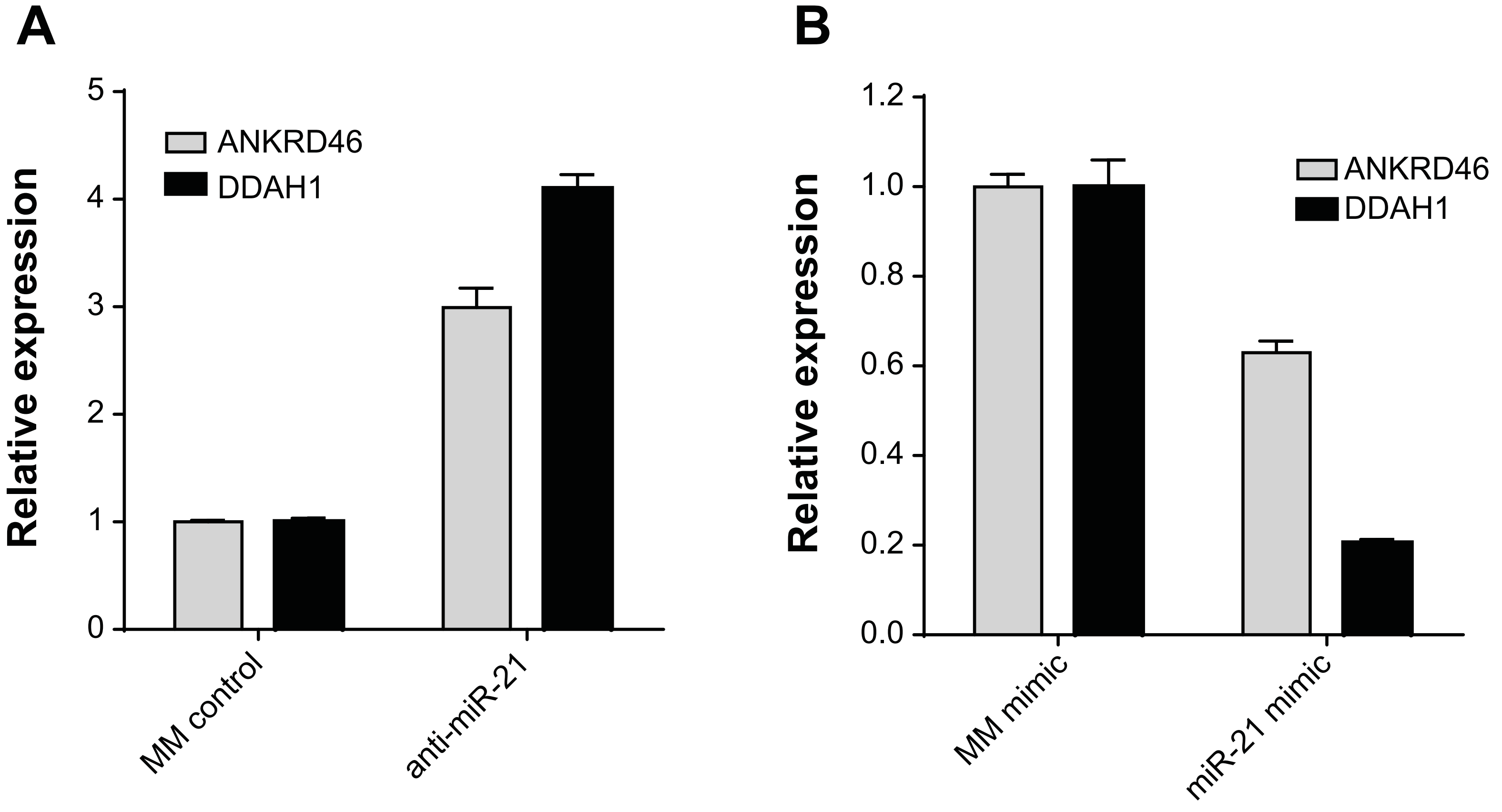

Supplementary Figure 6

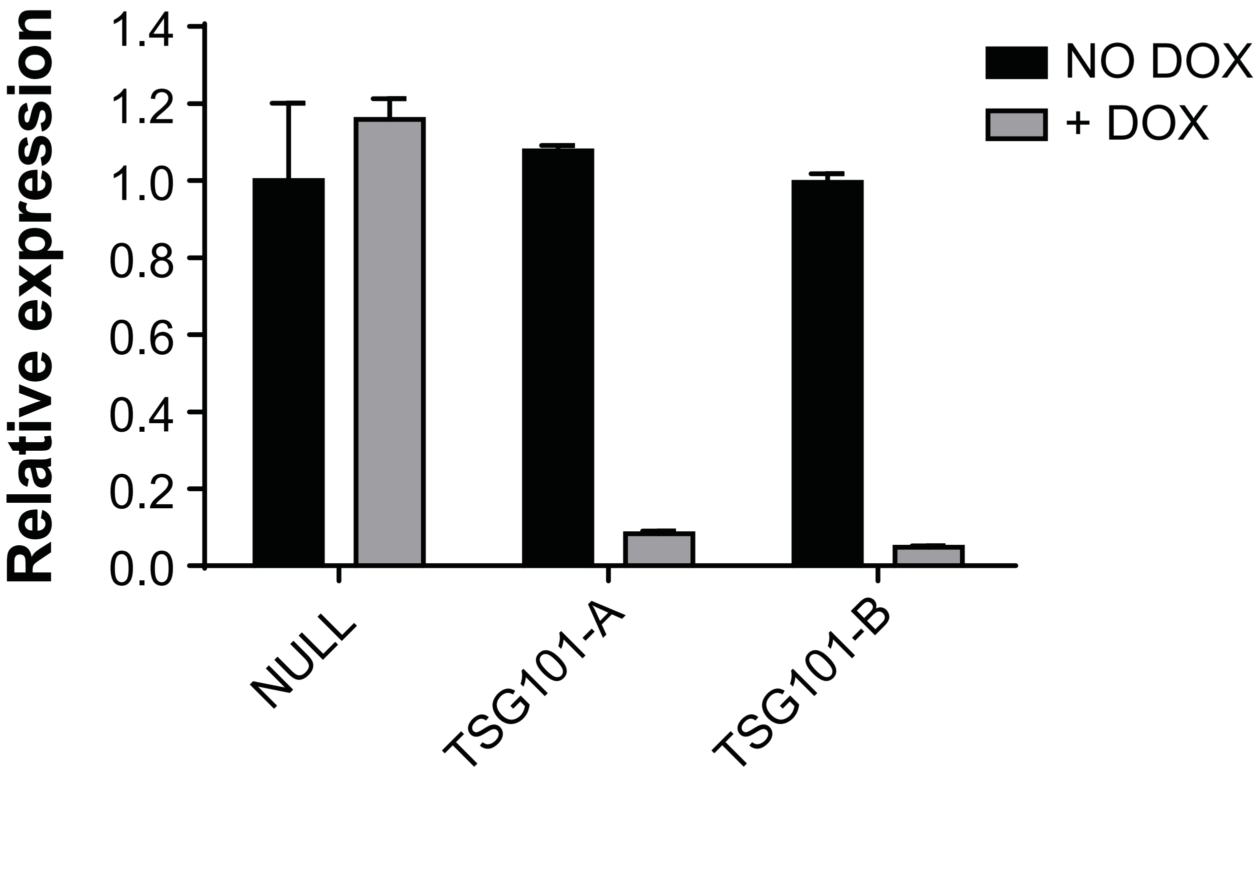

Supplement: SUPPLEMENTARY DATA [file supp_gku1367_nar-03084-y-2014-File009.pdf]
